# 3D-Ultrasonography for evaluation of facial muscles in patients with chronic facial palsy or defective healing: a pilot study

**DOI:** 10.1186/1472-6815-14-4

**Published:** 2014-04-25

**Authors:** Gerd Fabian Volk, Martin Pohlmann, Mira Finkensieper, Heather J Chalmers, Orlando Guntinas-Lichius

**Affiliations:** 1 Department of Otorhinolaryngology, Jena University Hospital, Lessingstrasse 2, D-07740 Jena, Germany; 2 Department of Clinical Sciences, Ontario Veterinary College, University of Guelph, 50 McGilvray St.Guelph, Guelph, ON N1G 2 W1, Canada

**Keywords:** 3D-Sonography, Facial musculature, Reconstructive surgery, Facial nerve, Facial palsy

## Abstract

**Background:**

While standardized methods are established to examine the pathway from motorcortex to the peripheral nerve in patients with facial palsy, a reliable method to evaluate the facial muscles in patients with long-term palsy for therapy planning is lacking.

**Methods:**

A 3D ultrasonographic (US) acquisition system driven by a motorized linear mover combined with conventional US probe was used to acquire 3D data sets of several facial muscles on both sides of the face in a healthy subject and seven patients with different types of unilateral degenerative facial nerve lesions.

**Results:**

The US results were correlated to the duration of palsy and the electromyography results. Consistent 3D US based volumetry through bilateral comparison was feasible for parts of the frontalis muscle, orbicularis oculi muscle, depressor anguli oris muscle, depressor labii inferioris muscle, and mentalis muscle. With the exception of the frontal muscle, the facial muscles volumes were much smaller on the palsy side (minimum: 3% for the depressor labii inferior muscle) than on the healthy side in patients with severe facial nerve lesion. In contrast, the frontal muscles did not show a side difference. In the two patients with defective healing after spontaneous regeneration a decrease in muscle volume was not seen. Synkinesis and hyperkinesis was even more correlated to muscle hypertrophy on the palsy compared with the healthy side.

**Conclusion:**

3D ultrasonography seems to be a promising tool for regional and quantitative evaluation of facial muscles in patients with facial palsy receiving a facial reconstructive surgery or conservative treatment.

## Background

Acute denervation of the facial muscles after a severe facial nerve lesion leads to facial palsy. Furthermore, long-term denervation causes loss of the resting tone accompanied by progressive facial muscle atrophy. Therefore, it is generally recognized that a longer duration of denervation is a negative prognostic factor for a good functional recovery after facial nerve reconstruction [1,2]. According to a general doctrine, facial nerve reconstruction surgery is not recommended beyond two to three years after degenerative facial nerve lesion [3]. Obviously, this rule of thumb is rough as the time course of muscle atrophy seems to be highly variable. Yet, a fast, non-invasive, and reliable method to evaluate the condition of the facial muscles and the degree of atrophy is missing. So far, the only method used in clinical routine is needle electromyography (EMG) [2,4]. The vitality of the muscles is assessed by subjective and qualitative examination of the insertion activity. Other EMG parameters, such as spontaneous muscle activity or evaulation of voluntary activity, which may be used to predict the degree of facial nerve degeneration, do not allow an evaluation of the degree of muscle atrophy.

Principally, one pilot study has shown that magnetic resonance imaging (MRI) is able to demonstrate facial muscles and to evaluate the degree of muscle atrophy by bilateral comparison [5]. Probably, specific MRI drawbacks have hindered a usage in clinical routine; namely there is restricted access to MRI, it is time consuming and costly. Furthermore, when facial MRI is elected, adjusting the sectional planes to the individual face requires a high practical knowledge. In order to optimize sequence acquisition for each muscle, multiple pulse sequences would be necessary. In contrast, ultrasonography allows individual cross-sections optimized for every muscle during real time imaging. It can also detect distinctive patterns in muscles affected by a neuromuscular disease [6]. Additionally, the spatial resolution of MRI is inferior to high frequency (8-15 MHz) ultrasound. Moreover, the additional application of three dimensional (3D) ultrasonography produces volumetric data that might simplify the quantification of muscle atrophy [7].

A first report on visualization of the muscles of facial expression with ultrasonography was published in 1988 [8]. Interestingly, although possibly attributable to the technical limitations in early generation ultrasound equipment, the possibilities of ultrasonography have not been further explored until recently [9]. In the present pilot study we explored the possibilities of modern 3D ultrasonography to allow a fast assessment of regional muscle volume changes in patients with facial palsy.

## Methods

### Subjects

To establish 3D ultrasonography of facial muscles, a healthy adult volunteer was recruited (#0). He never had a facial trauma, head trauma, facial palsy or any other neurological disorder. He had no history of a hereditary neuromuscular disorder or of any other congenital disorder. Afterwards, 3D ultrasonography was performed in a cross section of seven patients (#1-7) affected with a history of facial palsy of different etiology, duration, and treatment (Table 1). The study was approved by the local ethics committee, and informed consent was obtained from all participants. Age, gender, body weight, body height, body mass index (BMI), and handedness of all subjects were recorded. The local ethics committee of the Friedrich-Schiller University, Jena, Germany, approved the study. Informed consent including consent for publication of the report and any accompanying images was obtained from the volunteer and the patients.

**Table 1:** Patients’ characteristics

**Patient**	**Age (years)**	**Sex**	**Etiology**	**Interval since onset of palsy (months)**	**Type of reconstruction**	**Interval since reconstruction (months)**	**Side**	EMG results*
#1	32	M	Brainstem surgery for medulloblastoma	37	Selective HFJ	18	L	IA: reduced
								PSA: none
								VA: Reinnervation potentials in nasalis m., zygomatic m., and orbicularis oris m. during tongue movements
								I: Reinnervation lower face by hypoglossal nerve
#2	26	F	Unclear	432	HFJ	7	R	IA: reduced
								PSA: none
								VA: Reinnervation potentials only in orbicularis oris m. during tongue movement
								I: Reinnervation by hypoglossal nerve has started
#3	26	F	Vestibular schwannoma surgery	37	HFJ	2	L	IA: reduced
								PSA: none
								VA: none
								I: Reinnervation has not yet started
#4	63	F	Glomus jugulare tumor surgery	35	None	NA	L	IA: reduced
								PSA: none
								VA: none
								I: severe lesion without signs of spontaneous regeneration; HFJ planned for same day
#5	65	F	Glomus jugulare tumor surgery	10	None	NA	R	IA: normal
								PSA: none
								VA: none
								I: severe lesion without signs of spontaneous regeneration; HFJ planned for one week later
#6	39	F	Idiopathic palsy	23	None	NA	R	IA: normal
								PSA: none
								VA: reduced in zygomatic muscles, synkinetic activity between orbicularis oris and oculi m.
								I: defective healing with synkinesis
#7	46	F	Vestibular schwannoma surgery	87	None	NA	R	IA: normal
								PSA: none
								VA: synkinetic activity between orbicularis oris and oculi m., massive activity in orbicularis oculi m.
								I: defective healing with synkinesis and hyperkinesis

NA = not applicable; *Insertion activity (IA); Pathologic spontaneous activity (PSA); Voluntary activity (VA); Interpretation (I).

### Electromyography

Electromyography (EMG) was performed with a Medelec Synergy EMG system (Viasys CareFusion, Höchberg, Germany). The examination technique was published in detail elsewhere [10]. The paralyzed hemiface was examined at rest and during voluntary activity, and the function of six facial muscles was analyzed: the frontalis, orbicularis oculi, major zygomatic, orbicularis oris, levator labii superior, and the depressor anguli oris muscle. The EMG recordings were analyzed for insertion activity, pathologic spontaneous fibrillation potentials, the degree of voluntary polyphasic reinnervation potentials, and for synkinetic activity.

### 3D Ultrasonography

**Figure 1:**
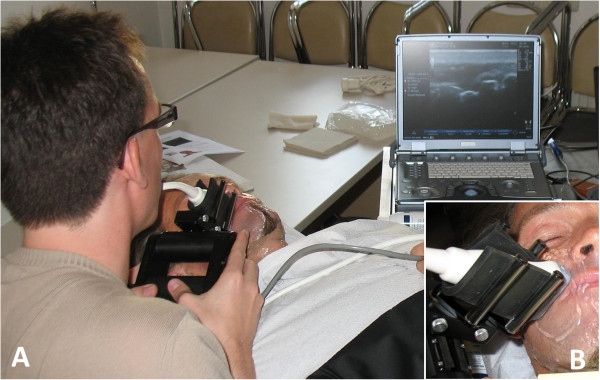
**3D ultrasonographic examination of facial muscles.** The linear array transducer was attached to a motorized 3D US acquisition system driven by a custom built linear mover connected to a laptop computer **(A)**. Details of the attachment of the transducer to the 3D acquisition system are shown in **(B)**.

The ultrasonography examinations (Figure 1) were performed by three of the present investigators (G.F.V.; M.P.; H.C). A GE Logic E ultrasound machine with a L12–5 Mhz linear array transducer was used for the composite imaging (GE Healthcare, Wisconsin, USA). The probe had an acoustic window of 38.5 mm. The probe was attached to a motorized 3D US acquisition system driven by a custom built linear mover (Robarts Institute, University of Western Ontario, Canada) with an Lenovo ThinkPad laptop computer (64 bit operating system 8 GB RAM, Inter core i5 CPU @ 2.4 GHz; Lenovo, North Carolina, USA). This was equipped with a frame grabber digitizing 2D frames at 30 Hz from the ultrasound machine as the probe was moved along the face at a uniform speed of 3 mm/s driven by the robotic mover along a maximum length of 10 cm. 3D volumes were constructed using the acquired frames and displayed using multiplanar reformatting and custom software (Robarts Institute, University of Western Ontario, Canada). To illustrate the measured muscle volumes, the 3D reconstructions were color-coded and superimposed on photographs of the faces of the patients.

### Identification of facial muscles

**Figure 2:**
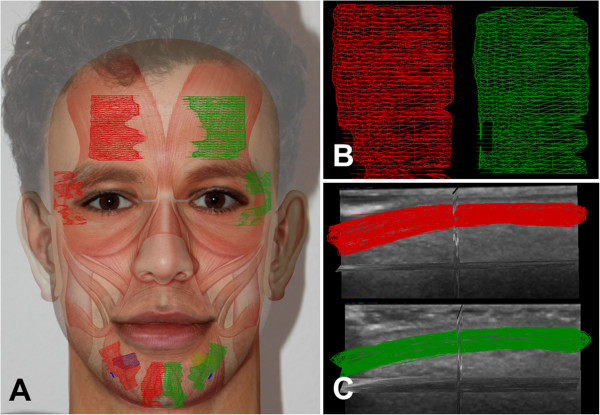
**Normal healthy human face.** Superimposition of facial muscles and measured areas of facial muscles **(A).** Close-up of 3D reconstruction of parts of right (red) and left (green) frontalis muscle in sagittal plane **(B)** and axial plane **(C)**.

The primary aim was to identify different facial muscles using the 3D system and to perform comparison between healthy and affected sides. All subjects were examined in supine position and completely relaxed. Before starting the 3D acquisition, volunteer activation of facial muscles was used to confirm the correct position of the transducer. The transducer was positioned perpendicular to the facial skin and acoustic coupling gel was used generously to facilitate the motorized movement of the probe and to minimize contact artifact at facial contours. A muscle was considered to be identifiable when it was possible to delineate the facial muscle throughout its complete course without interference from other radiating adjacent facial muscles (An example is provided in Figure 2: the frontalis muscle on both sides). The overlap with other mimic muscles with mutual anchoring is a characteristic feature of some mimic muscles. In these areas, a distinction of each mimic muscle was often impossible. This circumstance would skew the muscle area measurement. It must be stressed that the aim was not to measure the complete, absolute, volume of the mimic muscles but to measure the volume of the same part of each muscle through direct bilateral comparison. Therefore, the absolute measured volumes are not of interest but the relation between palsy side and healthy side.

Parts of five facial muscles were clearly delineated from surrounding connective tissue, bone, adjacent facial muscles, and were suitable for 3D ultrasonography (Figure 2A). In the cranio-caudal direction these muscles were: frontalis muscle, orbicularis oculi muscle, depressor anguli oris muscle, depressor labii inferioris muscle, and mentalis muscle. Because we only measured parts of the muscles, it was very important to measure always the same part of the muscle on both sides and in all subjects. Hence, reliable landmarks were very important: The frontal muscle was measured in two 60 mm long strip-like acoustic windows starting from the supraorbital margin going cranial. Vertical lines through the pupils were used to determine the midline of each strip. To standardize the acquisition of the orbicularis oculi muscle, a horizontal line through the pupils marked the middle of this cranio-caudal orientated 60 mm strip. The lateral wall of the orbital cavity and the frontal process of the zygomatic bone were important landmarks for this scan of the orbicularis oculi muscle.

The depressor anguli oris muscle and the depressor labii inferioris muscle were measured using one scan on each side. The alveolar processes of the mandibular body were used as landmarks for these scans, starting on a standardized axial position. From here, the line probe excursions were set for 30 mm both cranial and caudal. The mentalis muscles of both sides were measured using a single scan, orientated in the midline on the face. The lower lip was the cranial border, the mental protuberance the caudal one. In most patients, this scan was smaller than 60 mm.

### Statistics

All statistical analyses were performed using IBM SPSS, version 19.0. If not reported differently, data are presented as means ± standard deviation. The non-parametric Kruskal Wallis test for dependent parameters was used to compare muscle volumes between both facial sides. The ratio of the volume of each mimic muscle on the palsy in relation to the healthy side was calculated as follows: Ratio = Palsy side/healthy side (Table 2).

**Table 2:** Volumes of the same region of different mimic muscles on both sides of the face in a healthy subject* and seven patients

**Patient**	**Frontal muscle**			**Orbicularis oculi muscle**			**Depressor anguli oris m.**			**Depressor labii inf. muscle**			**Mentalis muscle**		
	**Partial volume (mm**^**3**^**)**			**Partial volume (mm**^**3**^**)**			**Partial volume (mm**^**3**^**)**			**Partial volume (mm**^**3**^**)**			**Partial volume (mm**^**3**^**)**		
	**Healthy side**	**Palsy side**	**Ratio**	**Healthy side**	**Palsy side**	**Ratio**	**Healthy side**	**Palsy side**	**Ratio**	**Healthy side**	**Palsy side**	**Ratio**	**Healthy side**	**Palsy side**	**Ratio**
#0	559.18	462.61	0.83	317.15	302.46	0.95	1143.79	1084.22	0.95	414.38	348.87	0.84	534.16	502.49	0.94
#1	430.05	512.75	1.19	248.13	31.24	0.13	590.94	208.01	0.35	281.57	7.95	0.03	388.84	147.30	0.38
#2	1489.67	963.28	0.65	116.31	32.34	0.28	418.77	82.26	0.20	142.29	15.67	0.11	225.57	104.83	0.46
#3	763.21	1025.27	1.34	159.94	41.66	0.26	537.21	114.63	0.21	165.53	27.76	0.17	239.04	154.60	0.65
#4	1605.73	1845.93	1.15	105.79	55.00	0.52	548.25	211.70	0.39	392.06	198.29	0.51	367.92	173.88	0.47
#5	1214.30	1290.93	1.06	212.49	11.35	0.05	NA	328.20	NA	NA	42.11	NA	371.95	227.44	0.61
#6	1747.60	1265.61	0.72	214.90	189.08	0.88	925.64	911.25	0.98	317.75	327.64	1.03	NA	NA	NA
#7	1317.75	1223.41	0.93	79.72	84.37	1.06	370.74	391.01	1.05	193.47	246.94	1.28	232.10	212.54	0.92

*In the healthy subject (#0), both facial sides are healthy. There is no palsy side in this subject. Ratio = Palsy side/healthy side, NA = not applicable.

## Results

### Patients

**Figure 3:**
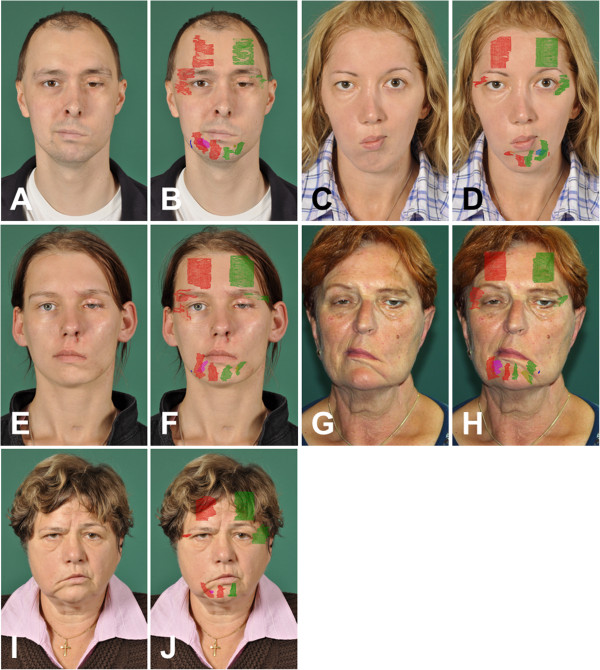
**Five patients with degenerative facial nerve lesion on the left side (AB, EF; GH) or right side (CD, IJ)) of the face; face at rest on the left side (A, C, E, G, I) and with projections of the muscle areas measured on both sides (B, D, F, H, J).** In two patients **(A-D)**, regeneration after reconstruction with hypoglossal facial nerve suture already started.

**Figure 4:**
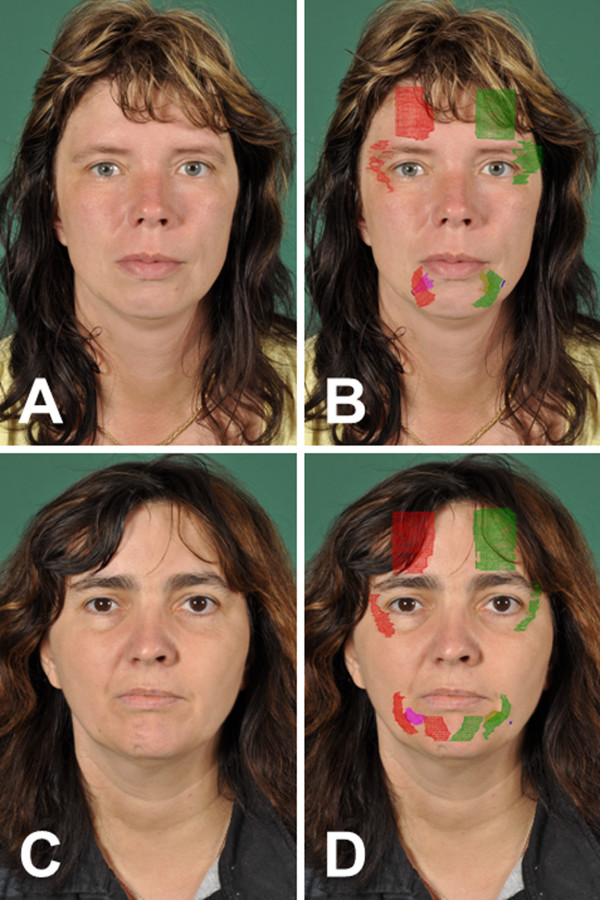
**Two patients with spontaneous defective healing after facial nerve lesion on the right side without reconstructive surgery.** Face at rest of both patients on the left side **(A, C)**, and same faces **(B, D)** with superimposed 3D reconstructions of the facial muscles.

Details on the seven patients with different types of facial palsy are presented in Table 1. Five patients (#1, #2, #3, #4, #5; Figure 3) presented 37 to 432 months after skull base or brainstem surgery with chronic facial palsy without any signs of regeneration. In three patients (#1, #2, #3; Figure 3A-F) reanimation of the affected hemiface was performed by hypoglossal-facial jump nerve suture (HFJ) 2 to 18 months before the 3D ultrasonographic examination. In the other two patients (#4, #5; Figure 3G-J), HFJ was performed one day (BI) and one week (BB) after the 3D ultrasonographic examination. Additionally, two patients (#6, #7; Figure 4A-D) with defective healing after spontaneous regeneration of severe facial palsy without surgical reconstruction were examined.

### Facial muscle volume on healthy side and facial palsy side in correlation to the EMG results

Projections of all measured muscle parts for all patients are presented in Figures 3 and 4. In one subject (#5), both depressor muscles were not completely visible on the healthy side. In another patient (#6), both mentalis muscle were not accessible due to a scar in this area.

An overview about the EMG results is given in Table 1. All US measurements are presented in Table 2. The healthy subject (#0) showed a right-left side relation of the facial muscle volumes between 83% and 94%. The fact, that all measured muscles on the right side were bigger than on the left side could be a hint for individual side-differences similar to handiness. As in the patients the measurements were robust and reproducible. Statistically, volume of the mentalis muscle and the orbicularis oculi muscle were significantly smaller on the palsy side in comparison to the healthy side in the patient prior to or during regeneration after facial nerve reconstruction (#1, #2, #3, #4, #5; p = 0.043, respectively). In the small sample, there was no side difference for the frontalis, depressor anguli oris, and for the depressor labii inferior muscle volume (all p >0.05). The frontal muscles did not show a side difference in size, even in patients without any sign of spontaneous regeneration (#4, #5). The minimum volume measured on the palsy was 3% of the healthy side for the depressor labii inferior muscle. These muscles also showed decreased insertion activity during the EMG examination but no pathologic spontaneous activity. The degree of muscle atrophy within different muscles in each of the five patients with severe facial nerve lesion was highly variable between the patients but also within the same patient (#1, #2, #3, #4, #5). In this small sample size a correlation of the degree of atrophy to the denervation interval or the regeneration interval (in patients #1, #2, #3 with facial nerve reconstruction) or to the result of voluntary EMG activity was not obvious. Even in the patient with a denervation time of 35 months and complete loss of resting tone (#4) who was evaluated in order to decide if facial nerve reconstruction still is indicated, at least half of the muscle volume still remained on the palsy side. This fact was co-decisive to indicate facial nerve reconstruction in spite of the long denervation of nearly three years. In the two patients with defective healing after spontaneous regeneration (#6, #7) a US volume side difference was not seen (all p >0.05). Interestingly, synkinesis and hyperkinesis (seen clinically and confirmed by EMG) were even more correlated to higher muscle volumes on the palsy than on the healthy side.

## Discussion

The clinical examination does not allow a reliable assessment of the vitality of the facial muscles in patients with long-term unilateral facial palsy. EMG is so far the only method available in clinical routine for an indirect assessment of extent of atrophy. But EMG does not allow a quantification of the degree of atrophy. Reduced or loss of insertion activity during needle EMG can be considered as a qualitative indication for muscle atrophy. For this reason, a more precise method would be of great value. From experimental data in other species, it appears that severe atrophy of the target musculature is an important negative prognostic factor for a good functional outcome in nerve reconstruction surgery [11,12]. As there is currently no better method to establish the argument for or against facial nerve reanimation, the recommendation to proceed is mainly based on the denervation time. Yet, there is no clear correlation between denervation time and atrophy of the muscles because other factors including site and type of the lesion, age, and type of conservative treatment may all influence the degree of muscle atrophy. Therefore, sometimes patients with longer denervation time show better functional results after facial nerve reconstruction than others with short denervation time [1].

In two retrospective MRI studies, asymmetry of the facial muscles was used to predict the outcome of facial nerve reconstruction and vestibular schwannoma surgery [5,13]. In both situations, pronounced asymmetry with reduced muscle mass on the affected side was associated with poor functional outcome. The facial muscles were evaluated on the MRI images as symmetrical or asymmetrical. The muscles that were evaluated were the zygomaticus, orbicularis oris and oculi, levator labii superioris, and nasalis muscles. One side was considered asymmetric compared with the other side when more than one muscle group was atrophied on consecutive images. The asymmetrical group was further qualitatively divided into mild or pronounced asymmetry, however data on the reliability of these assessments was not presented and objective measures of muscle size were not used. In contrast to the present study, no quantitative and reproducible measurements were performed in this MRI study.

As in the previous MRI studies, the present pilot study has shown that not all facial muscles can be assessed using ultrasound. Mimic muscles are often overlapping which hinders a distinct separation of the individual muscle bellies. Moreover, the size and stiffness of the probe with its attachment to motorized linear mover limit the access to some muscles over the surface contours of the face. This methodological problem will be overcome when smaller and more flexible systems are available. The major advantages of 3D US compared to MRI are the better accessibility, lower costs, individual planning of the section planes, the ease of repetition, and the fast potential to quantify muscle atrophy in bilateral comparison. As we could not measure complete muscle volumes, it is not possible to give absolute size of single muscles but a bilateral comparison is possible. Doing so, we can detect even small remnants of atrophic musculature: In one patient (#1), only 3% of the depressor labii inferior muscle was left on the paralyzed side in comparison to the healthy side. We can confirm that the degree of atrophy is highly variable between patients. For instance, patients #1 and #4 have the same denervation time, but although facial nerve regeneration takes place already in #1 after facial nerve reconstruction, the degree of atrophy is much more severe in some mimic muscles than in #4 who was waiting for reconstructive surgery. Furthermore, the pilot study revealed that actually in all patients the degree of atrophy is variable in-between mimic muscles of different facial regions in the same patients although the nerve was lesioned before its separation into the peripheral end branches. This might be relevant when regional reconstruction techniques or combinations of nerve surgery with regional static measures are discussed for an individual patient. Interestingly, we could detect in the two patients with defective healing (#6, #7) for the first time a hyperplasia of synkinetic muscles.

## Conclusion

Of course, the presented data is only preliminary. A larger sample size has to be measured and especially the major advantage of the 3D US technique has to be applied: Next step, we will perform serial measurements over time of the same muscles in patients with nerve surgery (here we expect an increase of muscle volume) and in patients under botulinum toxin treatment for synkinesis (here we might expect a decrease of muscle volume). In both situations, the 3D US might help us to monitor for the first time the time course of muscle gain or loss after therapeutic interventions in the face.

## Abbreviations

3D: Three dimensional; BMI: Body mass index; EMG: Electromyography; MRI: Magnetic resonance imaging; US: Ultrasound.

## Competing interests

There is no conflict of interest. The authors confirm that they do not have any financial relationship concerning this research.

## Authors’ contributions

GFV, OGL and HJC had the idea for the study. OGL drafted the manuscript. MP and MF performed the ultrasound at the participants. OGL performed the statistical analysis. OGL designed tables and figures. All authors read and approved the final manuscript.

## Pre-publication history

The pre-publication history for this paper can be accessed here:

http://www.biomedcentral.com/1472-6815/14/4/prepub
